# Effect of Gum Arabic karroo as a Water-Reducing Admixture in Concrete

**DOI:** 10.3390/ma9020080

**Published:** 2016-01-28

**Authors:** Rose Mbugua, Ramadhan Salim, Julius Ndambuki

**Affiliations:** Department of Civil Engineering, Tshwane University of Technology, Private Bag X680, Pretoria 0001, South Africa; SalimRW@tut.ac.za (R.S.); NdambukiJM@tut.ac.za (J.N.)

**Keywords:** Gum Acacia karroo, concrete, water-reducing admixture, compressive strength

## Abstract

Concrete is one of the most popular construction materials in the world. Chemical admixtures are ingredients added to concrete to enhance its properties. However, most chemical admixtures on the market today are expensive, thereby making them out of reach for small consumers of concrete. In Africa, use of chemical admixtures is rare despite the harsh weather conditions. In the current study, Gum from Acacia karroo (GAK) was used as a water-reducing admixture in concrete. A slump test, density and compressive strength were studied using different dosages of GAK while neat concrete was the control. Results showed that slump increased by 200% at a 2% dosage of GAK. This enabled reduction of water-to-binder (w/b) ratio from 0.61 to 0.48 for samples with a 3% dosage. Reduction in w/b resulted in increased compressive strength of 37.03% above the control after 180 days of curing for a 3% dosage. XRD studies also showed a decreased rate of hydration in the presence of GAK in concrete. It was concluded that GAK can be used in concrete as a water-reducing admixture, which is environmentally-friendly, thus producing sustainable and greener concrete.

## 1. Introduction

Concrete is a composite material made up of cement, aggregates, water, chemical admixtures and mineral admixtures. Use of chemical admixtures has grown considerably in the last four decades [1]. Although in Africa, the usage of admixtures is currently very low, it is expected to increase due to the increase in the construction of new infrastructure, such as new residential buildings, roads, bridges and water retention structures. South Africa has currently the highest per capita income in cement consumption in Africa and consequently concrete. However, use of chemical admixtures is still low and mainly limited to high value projects due to their high prices and lack of awareness in the construction industry. Furthermore, use of biodegradable materials in concrete is the trend today for sustainable and environmentally-friendly concrete. Thus, the use of natural admixtures to enhance the properties of concrete considering prevailing harsh weather conditions in Africa could lead to durable and cost-effective concrete.

Most admixtures are either organic or inorganic. Among the organic chemical admixtures used in concrete are natural gums. Gum arabic and tragacanth gum, which are natural gums, are good deflocculants, as well as good thickeners. They may have water-reducing properties and a dispersing effect and have been used as pumping aids [2]. These gums reduce compressive strength in concrete due to increased viscosity. Welan gum, which is in the same class as gum arabic, has been reported to increase cohesiveness by Lachemi *et al.* [3] and Rols *et al.* [4]. The mechanisms involved in increasing cohesiveness are either by water adsorption association or entanglement and association. Khayat [5] classified these gums as polysaccharides with high water retention properties. Apart from the natural gums, other admixtures, such as starch ether, which is a viscosity-modifying agent (VMA), lignosulfates and polycarborylate ether (superplasticizers), have been considered for use in Africa [6]. However, due to their derivation process, which make them expensive, they are still not accessible to medium and small concrete consumers. To reverse this, we propose use of Gum from Acacia karroo (GAK) in concrete as a water-reducing admixture.

GAK comes from Acacia karroo Haynes, which grows mainly in the southern countries of Africa (Zimbabwe, Mozambique, Zambia and Angola), while gum arabic (GA) comes from Acacia senegal or seyal species, which grow in countries in the north of Africa, like Sudan, Chad and Nigeria. Gum arabic is mostly referred to as gum from Acacia senegal and seyal and is mostly sought because of its purity and high grade [7]. GA is used primarily in the food industry for modifying the physical properties of foods and as a stabilizer. It is also used in the pharmaceutical industry, printing and in paint production. Gum Acacia karroo used to be exported as “CapeGum”, but not anymore. Although there is no reported use of GAK in concrete in the literature, there have been attempts to use gum arabic (GA) from Acacia seyal in concrete. Abdeljaleel *et al.* [8] reported increased compressive strength in concrete, while [9] used it to bond sand foundry molds.

Both gums (GAK and GA) contain different proportions of neutral sugars. They are made up of side chains of D-glucuronic acid with L-arabinose terminal units or L-rhamnose with a backbone made up of D-galactose units [10]. Gum Acacia karroo is a high molecular weight polysaccharide, $2.99\times 10^{6}$, that is slightly acidic. It is very soluble in water and can be used at different concentrations without being viscous.

Africa needs new a approach to the use of admixtures that have specific modification properties of concrete in addition to being compatible with African climatic conditions. For example, when concrete is mixed at elevated temperatures, there is a quick loss of workability due to high evaporation of mixing water, and the tendency is to add more water to the mix. Chemical admixtures can be used to increase workability without increasing the amount of mixing water. Thus, there is a need to develop admixtures that are natural, inexpensive and environmentally friendly. In this study, the influence of GAK on the mechanical properties of concrete was investigated. Concrete performance was determined by conducting a slump test, air entrainment and compressive strength at different water-to-binder(w/b) ratios and different GAK dosages. X-ray diffraction (XRD) analysis was carried out just to give an idea of hydration at 56 days.

## 2. Experimental Section

**Figure 1:**
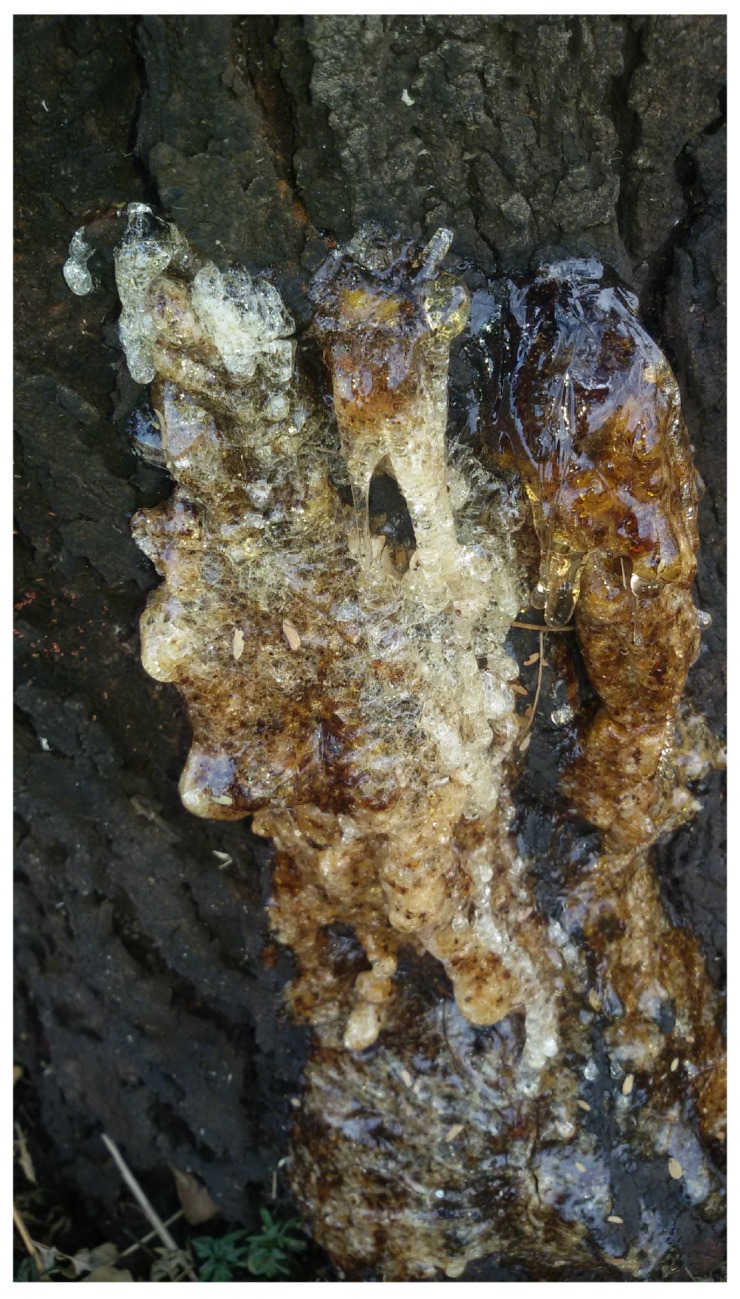
Gum karroo tears oozing from the bark.

Gum Acacia karroo (GAK) was picked from Pretoria Botanical Garden. The tears were hand-picked from the bark (Figure 1), cleaned by removing pieces of bark and any foreign matter and then dissolved in a part of gaugewater overnight. Polysaccharides are more efficient when pre-dissolved in water to enhance their rheological properties and water retention [11]. Soluble sugar content in GAK was determined using 80% ethanol and chloroform reagents.

Ordinary Portland cement CEM I (grade 52.5 MPa) blended with 25% unclassified fly ash was used to prepare all concrete specimens. All of the cement and fly ash was donated by Pretoria Portland Cement (PPC). X-ray fluorescence (XRF) was used to determine the composition of cement and the composition of fly ash. Potable water was used for mixing.

Crusher sand (CS) and coarse aggregates (CA) were both donated by Rosslyn Quarry in Pretoria. The fine aggregate conformed to SANS1083: 2006 [12]. The coarse aggregate used was of a nominal size of 19 mm and conformed to SANS 1083: 2006 [12].

**Figure 2:**
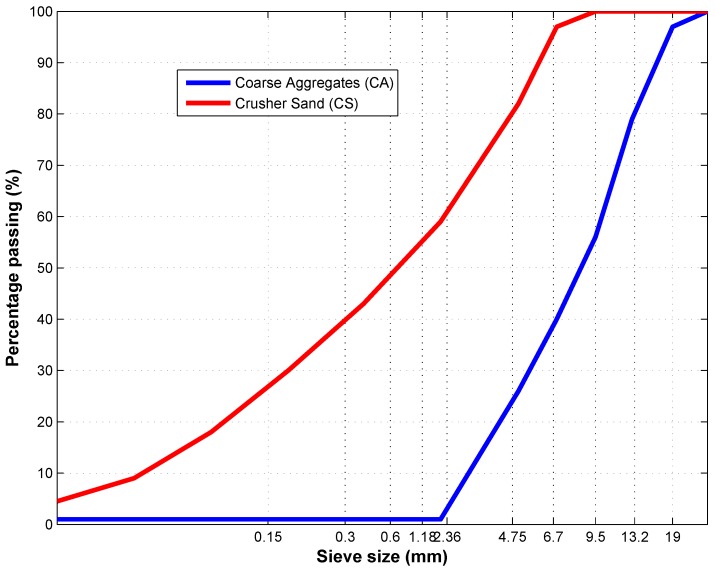
Grading curves for fine and coarse aggregates.

The fineness modulus (FM) of sand was 3.2, which fell within 2.9–3.5, which is categorized as coarse sand fineness [13]. Grading results for fine and coarse aggregates are shown in Figure 2.

Table 1 shows the cement and fly ash composition.

**Table 1:** Cement and fly ash composition.

Constituents	Cement (g per 100 g)	Fly Ash (Percentage by Mass)
SiO${}_{2}$	20.17	41.7
Al${}_{2}$O${}_{3}$	3.96	22.3
Fe${}_{2}$O${}_{3}$	2.35	2.4
Mn${}_{2}$O${}_{3}$	0.753	-
TiO${}_{2}$	0.29	-
CaO	60.57	18.2
MgO	2.71	1.8
P${}_{2}$O${}_{5}$	0.09	-
Cl	96.8 ppm	-
SiO${}_{3}$	2.39	-
Na${}_{2}$O	0.148	0.2
K${}_{2}$O	0.37	0.2
CaO${}_{2}$	1.50	-
SO${}_{3}$	1.5	-
Density (g/cm${}^{3}$)	3.12	-
Blaine surface (cm${}^{2}$/g)	3240	-

### 2.1. Testing Procedures

The slump test was carried out according to SANS 5863-3: 2006 [14] immediately after mixing concrete. Mix proportions of concrete ingredients, namely GAK, cement, fly ash (FA), crusher sand (CS), coarse aggregates (CA) and water, are shown in Table 2.

Concrete density was measured at the age of testing, by weighing all of the specimens after curing, using an electronic weighing balance before pressing the cubes for the compressive strength test. The density of each specimen was calculated by dividing the weight of the cubes by the volume of molds.

The pressure method was used to measure the air content in concrete as per EN 12350-7: 2009 [15].

The compressive strength test was carried out according to SANS 5863: 2006 [16]. The design strength was 35 MPa at 28 days. Concrete cubes were cast using metallic molds measuring 100 × 100 × 100 mm. In the first step, the mixes prepared had a constant w/b of 0.61, while different GAK dosage levels of 0.3%, 0.5%, 0.7%, 0.8%, 1.0%, 2.0% and 3.0% weight of cement were incorporated in the mixes. In the second step, the w/b ratio at higher dosage levels (0.7%, 0.8%, 1.0%, 2.0% and 3.0%) was reduced by reducing the water content so as to keep the slump constant at 60–45 mm. Other ingredients proportions remained constant. The mix without GAK was used as the control. The average compressive strength for the three specimens was recorded to the nearest 0.5 MPa. Samples were tested at 3, 7, 28, 56, 120 and 180 days of age.

Accelerated carbonation was carried out in a carbonation chamber as per the method previously used by Gonen *et al.* [17]. The samples used for carbonation were prepared and cured for 28 days, after which they were surface dried. Half of the cubes prepared measuring 100 × 100 × 100 mm were coated with epoxy paint on four surfaces and left in the laboratory to dry for 24 h, while the other half were left without painting. Painting on four surfaces ensured that carbonation could only progress through two opposite sides. All samples were then placed in a humidity chamber at a temperature of 20 ± 2 ${}^{\circ }$C and at a humidity of 65% ± 5%. The samples were then exposed to 4% carbon dioxide for 28 days. Each cube was split into two equal halves parallel to the plane of the uncoated surface and sprayed with phenolphthalein. Readings were taken at two points of each half at the unpainted surface and an average of two sides recorded. The uncoated samples were tested for compressive strength.

Four samples with high dosages of GAK (1%, 2% and 3% dosage and reduced w/b of 0.56, 0.52 and 0.48, respectively) were prepared for XRD analysis. The aim of this analysis was to identify phases of hydration products at high dosages. The samples were analyzed with a PANalytical Empyrean diffractometer with a PIXcel detector and fixed slits with Fe filtered Co-K*α* radiation. The phases were identified using X’Pert Highscore plus software. The relative phase amounts (weight %) were estimated using the Rietveld method (X’Pert Highscore Plus software).

## 3. Results and Discussion

### 3.1. Material Properties

Precipitate from reagents for testing GAK contained 70% sugars, like fructose, glucose and sucrose, and its pH in soluble form was 4.5.

### 3.2. Slump Test

**Figure 3:**
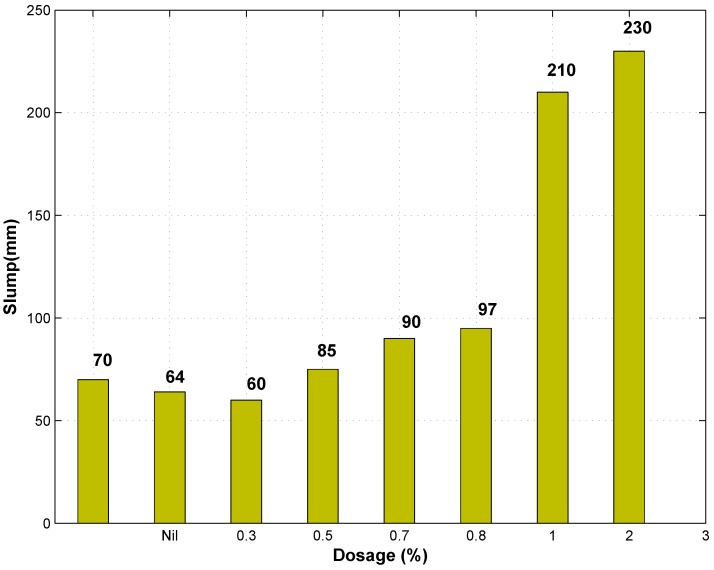
Slump of concrete with different dosages of Gum from Acacia karroo (GAK).

**Figure 4:**
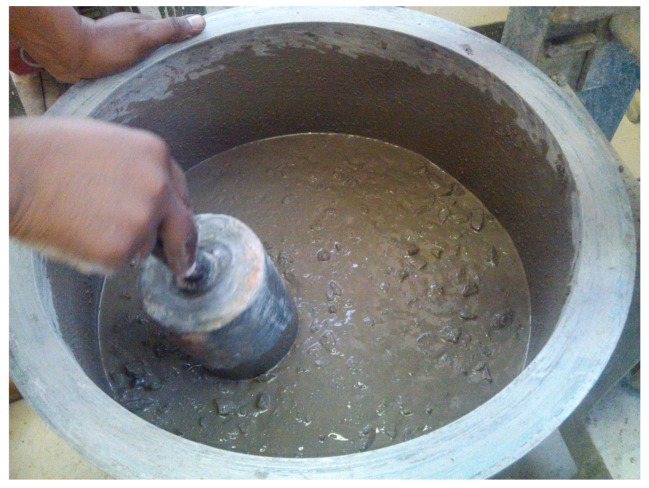
Concrete after the addition of a 2% GAK dosage.

Slump test results for concrete with different dosages of GAK are shown in Table 2, including the standard deviation (SD) of the slump test measurements. At a low dosage of GAK, the slump value remained almost the same as the control. At a 0.3% and a 0.5% dosage, there was a slight decrease in the slump of 8.5% and 14.2%, respectively, compared to the control, as shown in Figure 3. At a 0.7% dosage level, the value of slump increased by 21.4% above the control. This ensured that the concrete was still workable. At this dosage, an increase in workability was observed as the mixture became more watery, as shown in Figure 4. At 2% and 3%, the dosage slump increased by 200% and 222% compared to the control. Other researchers who reported an increase in workability while using polysaccharide gums are [18,19,20,21]. It can therefore be inferred that GAK probably caused dispersion of smaller cement grains to deflocculate and increased fluidity in the concrete mix either by repulsion of polymer absorption or by the zeta potential [22] due to its sugar content.

**Table 2:** Concrete mix proportions and fresh state properties. FA, fly ash; CS, crusher sand; CA, coarse aggregates.

GAK Dosage	Cement (kg)	FA (kg)	CS (kg)	CA (kg)	Water (kg)	w/b	Slump (mm)	SD Slump
Control	293	93	903	1190	241	0.6	70	5
0.3%	293	93	903	1190	241	0.6	64	6
0.5%	293	93	903	1190	241	0.6	60	8
0.7%	293	93	903	1190	241	0.6	85	4
0.8%	293	93	903	1190	241	0.6	90	3
1.0%	293	93	903	1190	241	0.6	97	4
2.0%	293	93	903	1190	241	0.6	210	1
3.0%	293	93	903	1190	241	0.6	230	2
0.7%	293	93	903	1190	196	0.5	50	8
0.8%	293	98	903	241	196	0.5	55	9
1.0%	293	98	903	216	220	0.56	58	8
2.0%	293	98	903	206	206	0.52	45	1
3.0%	293	98	903	187	187	0.48	60	2

As the dosage increased, the effect of deflocculation of cement grains by GAK is seen by increased slump values. Increased fluidity in concrete in the presence of GAK can be explained by the fact that cement particles tend to form clusters, which trap water within them in the absence of water reducers. An increase in the fluidity of concrete in the presence of high dosages of GAK could be due to the break up of the clusters and maintaining dispersion, so that each particle exists as an individual. This results in increased mobility of the fresh concrete [23]. Fluidity increases due to increased adsorption of admixture onto the surface of cement, allowing cement grains to be well distributed in the mix. The homogeneous suspension of particles and reduced inter-particle collusion reduce both bleeding and internal stress, making concrete more flowable.

Mixes with GAK and a reduced w/b ratio lost slump after approximately 10 min of mixing, thereby requiring more effort for compaction and reducing the compaction time. This is due to the adhesive nature of GAK, which made the particles stick together, resulting in a stiff concrete mix. Loss of slump could have been probably caused by the amount of gypsum and the tri-calcium-aluminate in cement [24]. This can be controlled by re-dosing or using cement with low amounts of gypsum and tri-calcium-aluminate.

### 3.3. Density

Values of mean density (plus their standard deviation) of concrete samples with different dosages of GAK are shown in Table 3. The increase in GAK dosage decreased the density values marginally. However, extended curing and reducing the w/b ratio generally increased the density. Reducing the w/b ratio from 0.61 to 0.5 at a 0.7% dosage increased density by 1.9% at 28 days. An increase of 1.9% was observed at the 1% level dosage when w/b was reduced from 0.61 to 0.56 at 56 days. A marginal increase in density can be related to an increase in compressive strength when the w/b ratio is reduced. The values obtained were within the density values of normal concrete, which lie between 2400 and 2600 kg/m${}^{3}$ [25].

**Table 3:** Mean density (×10${}^{3}$ kg/m${}^{3}$) of concrete samples with different dosages of GAK.

Dosage	Curing Age (Days)						w/b
	3	7	28	56	120	180	
0	2.462	2.476	2.47	2.464	2.485	2.482	0.61
SD	0.03	0.01	0.02	0.02	0.02	0.02	
0.3	2.512	2.463	2.484	2.478	2.454	2.463	0.61
SD	0.11	0.03	0.02	0.004	0.02	0.004	
0.5	2.448	2.45	2.447	2.451	2.454	2.482	0.61
SD	0.04	0.02	0.02	0.04	0.03	0.03	
0.7	2.436	2.438	2.441	2.442	2.448	2.456	0.61
SD	0.001	0.004	0.01	0.04	0.03	0.01	
1	2.439	2.441	2.44	2.444	2.443	2.498	0.61
SD	0.01	0.01	0.05	0.01	0.01	0.01	
0.7	2.449	2.475	2.49	2.46	2.5	2.47	0.5
SD	0.01	0.02	0.01	0.005	0.05	0.02	
0.8	2.464	2.469	2.51	2.5	2.51	2.5	0.5
SD	0.02	0.01	0.02	0.06	0.01	0.02	
1	2.434	2.422	2.461	2.492	2.534	2.512	0.56

### 3.4. Air Entrainment

**Figure 5:**
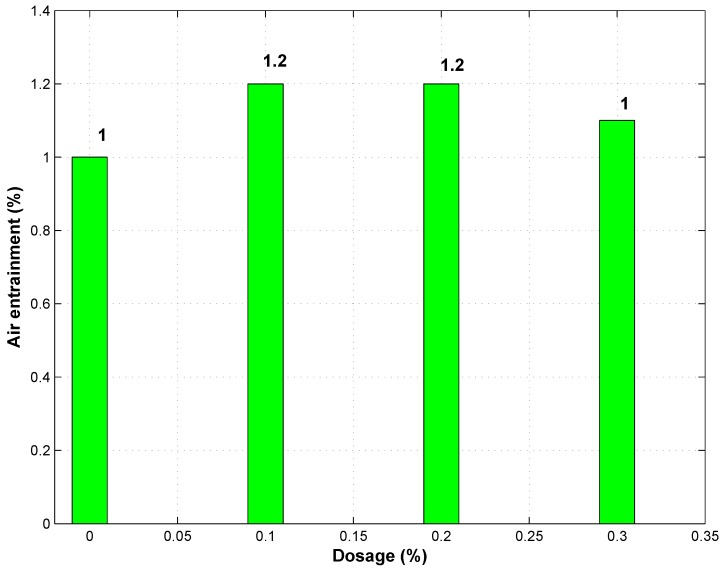
Percentage of air entrainment of concrete samples with GAK.

Most polysaccharide gums entrain air according to Wada *et al.* [26], Chandra *et al.* [27] and Laziniewska-Piekarczyk [28]. This was in agreement with the current study, where results indicated a 20% increase in air entrainment above the control for mixtures having a 0.5% and a 0.7% level dosage of GAK, as shown in Figure 5. However, there was no significant difference in entrained air between the 0.5% dosage and the 0.7% dosage. It was further noted that at the 1% GAK level dosage, there was a drop in the amount of entrained air by 8% compared to samples with 0.5% and 0.7% GAK, but 10% more than the control. Air entrainment was evident from the sample surface as more pores were observed on samples with GAK than the control.

Different dosages of GAK did not change the amount of air entrained significantly. This result was not expected to a certain extent due to the fact that there was a substantial decrease in strength with the increase in dosage. According to [29], some admixtures are said not to affect the fluidity after a certain dosage, and air entrainment content remains constant. At a 1% dosage, concrete was more liquid, and therefore, less air was entrained. The higher the GAK dosage, the less compressive strength and the higher the air entrainment. Smaller and less pores were observed on samples without GAK, while larger and more air pores were observed on the surface of samples with a higher dosage of GAK. Entrained air caused a reduction in density, which in turn reduced compressive strength.

### 3.5. Compressive Strength

**Figure 6:**
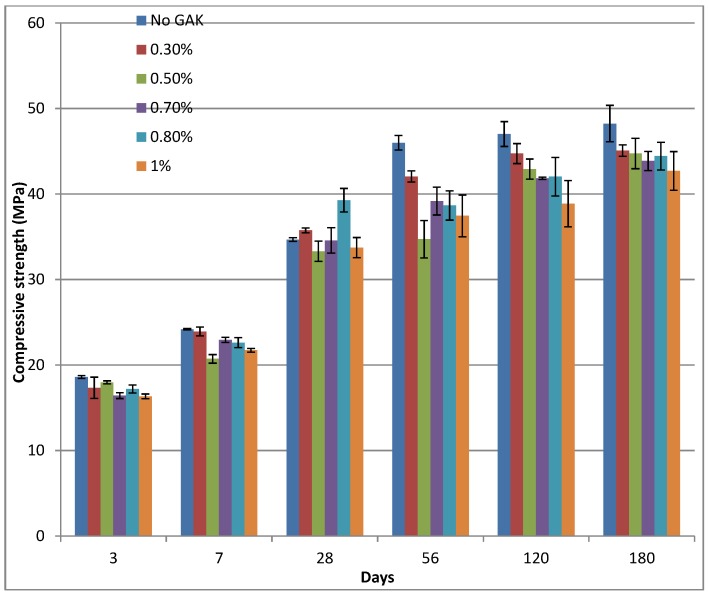
Compressive strength of concrete with different dosages of GAK and a water-binderratio (w/b) = 0.6.

Compressive strength results at different curing ages and different GAK dosages are shown in Figure 6 for samples with a 0.61 w/b ratio. At low level dosages of 0.3%–0.5%, there was little change in both workability and compressive strength. This was probably due to the fact that low dosages of GAK were not enough to affect the properties of the concrete. An increase in compressive strength was observed for all samples as curing period increased. As the dosage of GAK increased, compressive strength decreased compared to the control. As the GAK dosage was increased, the compressive strength decreased, but at a 0.7% and a 0.8% dosage, strength increased probably due to the fact that this was the optimum dosage, and a higher rate of hydration took place at 28 and 56 days. At 56 days of curing, a 17.5% difference in compressive strength was observed between the 0.3% and 0.5% GAK dosages. This gap closes to 0.7% at 180 days. Extended curing (after 180 days) closed the gap in compressive strength for samples with a 0.5, 0.7 and 0.8% dosage, as shown in Figure 6. Prolonged curing up to 180 days improved the compressive strength of samples with a 0.61 w/b ratio. However, the strength did not reach the value of the control. Compressive strength at this age for samples with GAK dosage levels of 0.5%, 0.7%, 0.8% and 1% was 7.3%, 9%, 7.9% and 11.5%, respectively, less than the control. All samples treated with GAK at a w/b ratio of 0.61 yielded concrete with a compressive strength below the design mix of 34 MPa. Nevertheless, the difference in values between samples with different dosages was less than 5%. This could be due to less effect of GAK at later age. This is probably explanation due to the reduction in the rate of cement hydration caused by the addition of GAK, which reduced the dissolution of alkalis in the pore fluid [30]. Other researchers reported a decrease in compressive strength when using polysaccharide gums [21,31].

**Figure 7:**
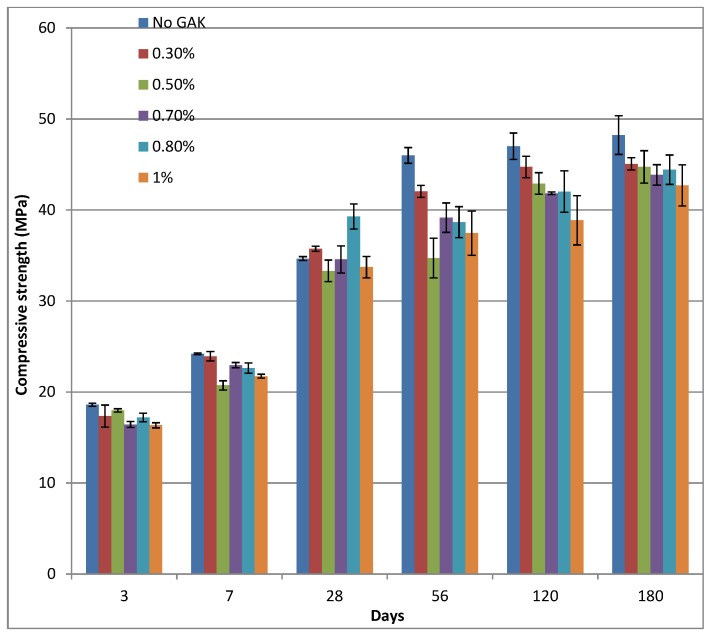
Compressive strength of concrete with different dosages of GAK and different w/b ratios.

**Figure 8:**
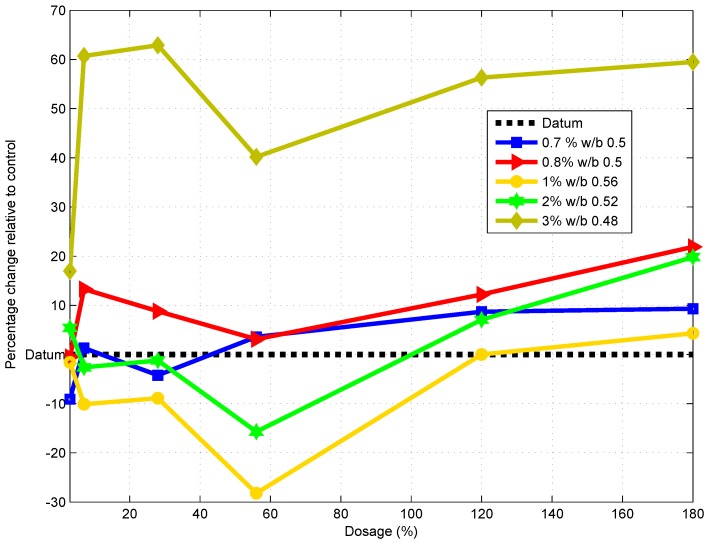
Percentage change in compressive strength with reduced water compared to the control.

Comparison of the mean compressive strength of the control with the compressive strength of samples with a reduced w/b ratio showed an increase in strength (see Figure 7), which indicates a change in strength relative to the control. Standard deviation data are also indicated. The highest percentage decrease for all samples was realized at the age of 56 days. At 56 days, samples with a 0.5%, 0.7%, 0.8% and 1% dosage had a decrease in strength of 24.5%, 14%, 15.9% and 18.6%, respectively, relative to the control (Figure 8). On the other hand, after 56 days of curing, there was a sharp increase in strength (over 20%) for the mix with 0.8% GAK and a reduced w/b ratio of 0.5. Reducing the w/b ratio from 0.61 to 0.52 and 0.48 for 2% and 3% level dosages increased compressive strength by 7.4% and 62.8%, respectively, relative to the control at 28 days of age and 20.2% and 59.5%, respectively, after 180 days of curing.

At a 1% dosage, the compressive strength with a w/b of 0.56 was less than that of the control at 7, 28 and 56 days, but higher by 4.3% than the control at 180 days. This phenomena could be explained by the reduced hydration rate at an early age due to the presence of GAK. The highest compressive strength was achieved at a 3% dosage of GAK with a w/b ratio of 0.48 relative to the control. Such high strength concrete is usually achieved by increasing the amount of cement. In this research, high strength concrete was achieved by using the same cement content, but reducing the w/b ratio by reducing the water content.

The water-binderratio has a critical impact on the concrete strength characteristic. For the proper chemical reaction to take place, minimum water (about 3% of mixing water) is required, while extra water increases the slump and reduces the strength. Excess water produces concrete with higher capillary porosity. Thus, water-reducing admixtures release the water trapped within the cement grains, thereby availing more water to participate in hydration. In the construction industry, a low w/b ratio is used to achieve concrete with high strength and high quality. However, this is not achievable without the use of admixtures if concrete is to remain workable, free from cracking and excessive shrinkage. Therefore, use of GAK as a natural plasticizer is beneficial to the construction industry, where high performance concrete is required. The increased compressive strength of samples with a reduced w/b ratio was as a result of the water reducing power of GAK while keeping concrete workable.

Concrete with a reduced w/b ratio develops strength at a higher rate than concrete at a higher w/b ratio. This is an important factor, since it is possible to remove formwork earlier, saving time during construction. The other important benefit is saving cement while achieving concrete of high strength. In most cases, when concrete of high strength is required, cement content is increased. Thus, reducing the amount of cement used is important for the environment, as well as reducing the cost of concrete.

### 3.6. Carbonation

**Figure 9:**
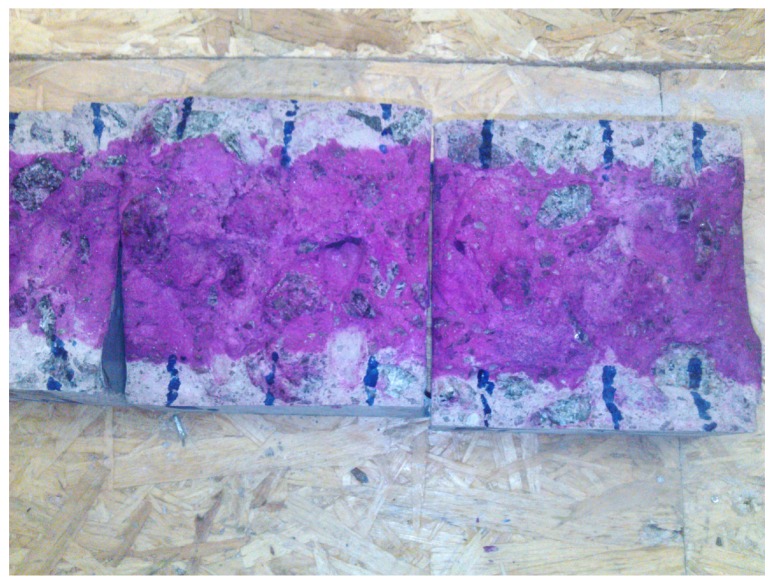
Samples after carbonation.

**Figure 10:**
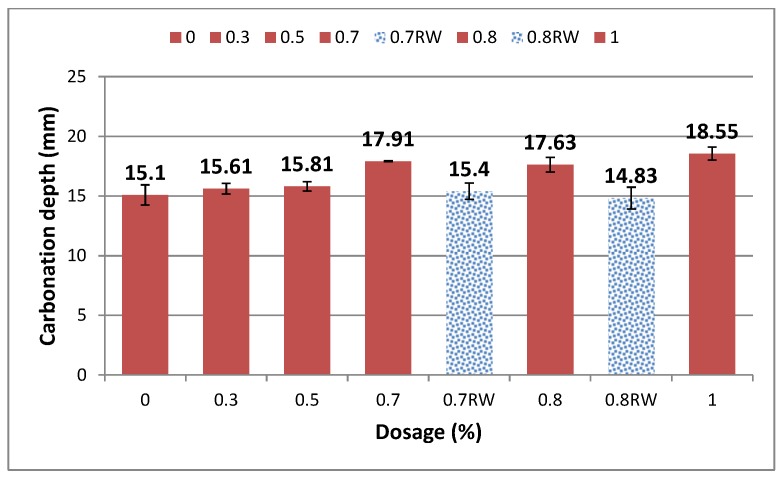
Carbonation of concrete samples.

The carbonated part of the specimen had no coloration, while the carbonated part turned purple due to the high alkalinity (Figure 9). It was observed that carbonation depth increased with the increase in GAK dosage when compared to the control. Carbonation results after 28 days of curing are presented in Figure 10. Carbonation of concrete with 0.3%, 0.5%, 0.7%, 0.8% and 1% dosages increased by 3.2%, 4.7%, 18.6%, 16.7% and 22.8%, respectively, compared to the control. Samples with a reduced w/b ratio of 0.5 and GAK dosages of 0.7 and 0.8% exhibited a decreased carbonation depth of 3.7% and 6% compared to the control. Comparing the carbonation depth between the w/b ratio of 0.61 and 0.5 at optimum dosages of 0.7% and 0.8% indicated a 18.4% decrease at a 0.7% dosage and 19.45% at a 0.8% dosage.

The carbonation rate depends on the concentration of CaO${}_{2}$, temperature, relative humidity and penetration pressure. In the current study, all of the above were kept constant, and only the dosage and w/b ratio of the specimen were changed. The increase in carbonation depth for the specimen with GAK can be explained by the increased presence of calcium hydroxide (CH) in the hydrated concrete due to the retardation of the hydration reaction.

Furthermore, the entrained air could also play a role in carbonation by increasing the transportation mode of the gas. The increase in carbonation depth could have been associated with less denser and lighter concrete. These results were confirmed by reduced compressive strength after carbonation, as shown in Table 4. A decrease in compressive strength after carbonation due to the presence of sucrose was also reported by [32]. Samples with a reduced w/b ratio exhibited less carbonation depth due the denser paste formation. Carbonation depth decreased with decreased w/b ratio. Improved carbonation with a lower w/b ratio was also confirmed by [33,34].

**Table 4:** Carbonation depth and compressive strength after carbonation.

	Test	
Samples	Depth (mm)	Comp.Strength (28 Days)
Control	15.1	49.85
0.3	15.61	48.63
0.5	15.81	47.91
0.7	17.91	45.44
0.8	17.63	46.2
1.0	18.5	41.81
0.7 RW	14.54	50.67
0.8 RW	14.2	49.44

### 3.7. XRD Studies

**Figure 11:**
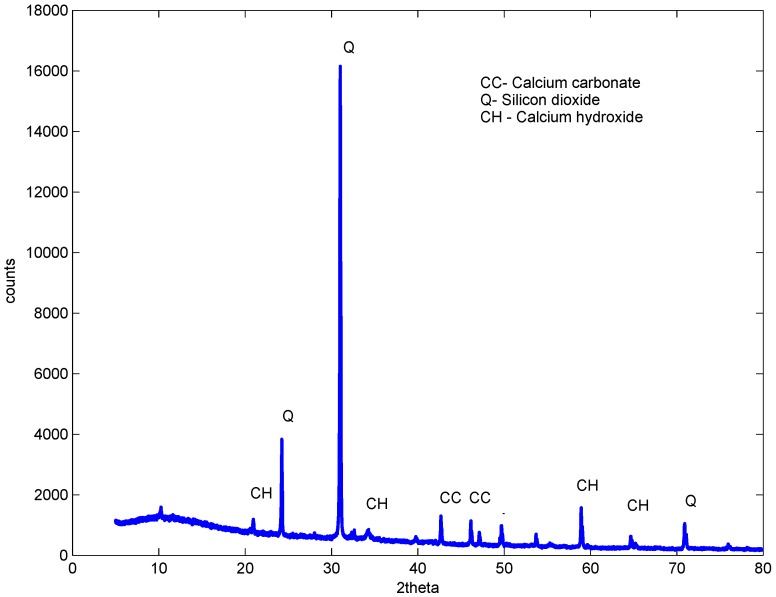
No GAK XRD sample at 56 days.

**Figure 12:**
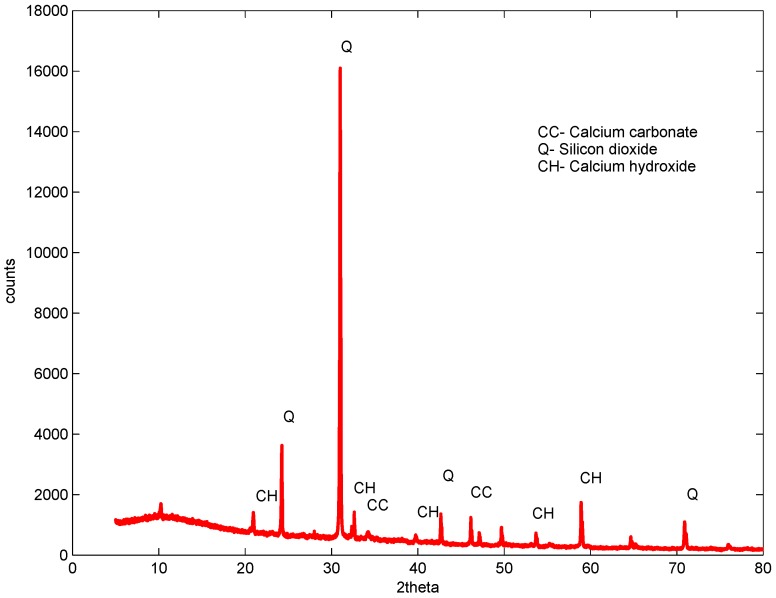
One percent dosage XRD sample at 56 days w/b = 0.56.

**Figure 13:**
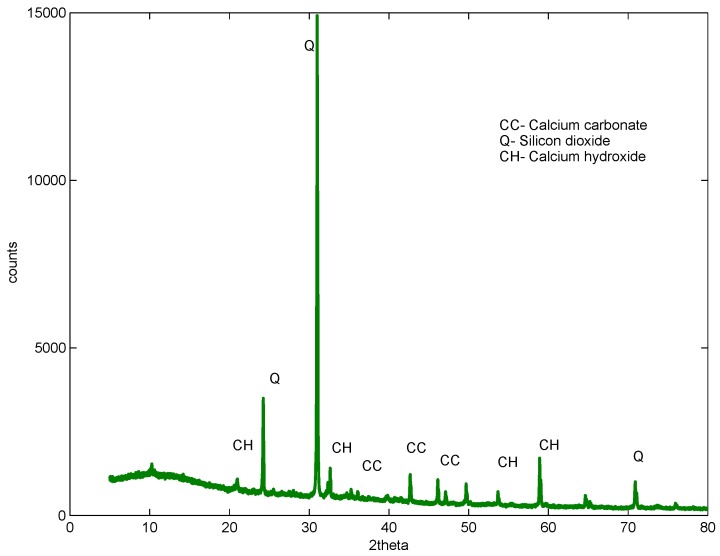
Two percent dosage XRD sample at 56 days w/b = 0.56.

**Figure 14:**
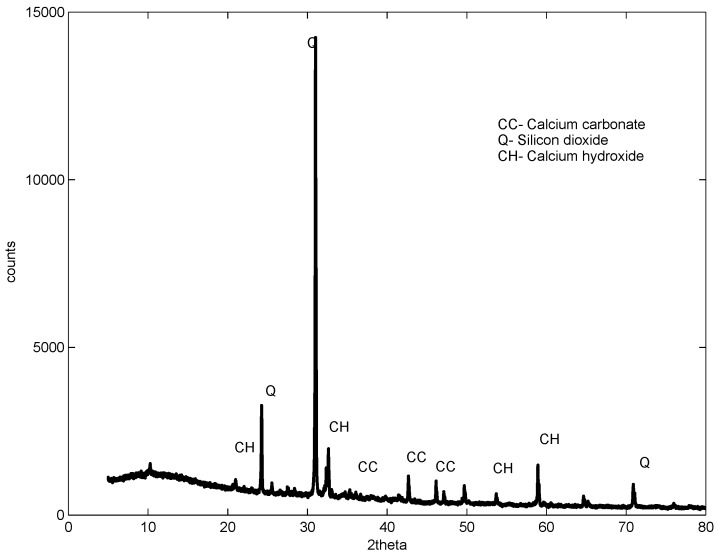
Three percent dosage XRD sample at 56 days w/b = 0.56.

XRD studies were carried out on samples after 56 days of curing. The control, the sample with a 1% dosage and a w/b ratio of 0.61 and two samples containing 2% and 3%, but with a w/b ratio of 0.52 and 0.48, were studied. The studies revealed almost a similar pattern for all samples with and without GAK, but with different intensities, as shown in Figure 11, Figure 12, Figure 13 and Figure 14. The analysis showed that there were no new chemical reactions during hydration and no existence of new chemicals. Comparing the hydration products formed, Rietveld analysis showed that a high percentage of calcium hydroxide (CH) was contained in the sample with a 1% dosage. At a 3% dosage and a w/b ratio of 0.48, there was a decrease in calcium hydroxide (CH) content, as shown in Table 5. This is an indication of the higher rate of hydration with a higher dosage of GAK and a lower w/b ratio. A decrease in compressive strength when GAK was added to concrete at a high w/b ratio can be attributed to the slow hydration rate, which reduces the gain of strength, especially in early ages. The decrease in strength due to slow hydration has previously been reported [35,36]. However, it was only the 3% dosage with a w/b ratio that showed improved hydration compared to the control.

The delayed hydration observed was as a result of calcium ions forming glucosate and sucroate in the presence of glucose and sucrose, respectively [37]. The formed compounds retard hydration by preventing cement grains from accessing water. The retarding effect was evident up to 56 days (Figure 8) after which all of the mixes started to gain strength. Use of polysaccharides may delay the formation of ettringite, as well as delay the usage of gypsum by glucose. Retardation may also depend on the content of C${}_{3}$A in cement [38].

**Table 5:** The relative phase amounts (weight %).

Sample	Quartz	Calcite	Portlandite
Control	83.67	4.25	2.29
3% RW	76.73	0.74	1.73
2% RW	82.85	0	2.32
1%	84.28	2.49	3.25

## 4. Conclusions

The effect of GAK use in concrete was tested, and different properties were studied both in the fresh and the hardened state. From the results obtained, the following conclusions were made:

(1) In hot weather climates, there is a tendency to add water to concrete to make it more workable. The addition of GAK to concrete can reduce this tendency. There was a remarkable improvement of concrete workability when GAK was used at above a 0.5% dosage. At a 2% dosage, the slump of concrete increased by 200%. However, the drawback observed was the quick slump loss due to the adhesive nature of GAK. This type of concrete is applicable in pre-cast technology.
(2) The increase in GAK dosage reduced the compressive strength and density of concrete. The air content also increased with increased dosage, which could have contributed to the compressive strength reduction. The highest decrease in compressive strength observed was 24.5% lower than the control at a 0.5% GAK dosage. In the case of a reduced w/b ratio from 0.61 to 0.48, the highest strength was observed at 3% GAK at all ages. The strength gain at 180 days was 37.03% higher than the control. The use of GAK as a water-reducing admixture is a possibility. Increased strength means GAK can be used as a water-reducing admixture, resulting in the reduction of cement demand, which in turn reduces carbon emissions and energy used, thereby providing the construction industry with a cleaner, greener and environmentally-friendly admixture. In addition, the gain of strength at an early age means formwork can be removed earlier, thereby decreasing construction time.
(3) The carbonation depth of concrete slightly increased with increased GAK dosage. The highest carbonation depth increased by 22.8% at a 1% dosage above the control. Samples with a reduced w/b ratio showed a decrease in carbonation.
(4) The XRD analysis gave an idea of the effect of GAK on hydration. The addition of GAK at higher dosages enables lower w/b ratios. This, in turn, showed an improved hydration rate relative to the control.
